# On Water Arrangements in Right- and Left-Handed DNA Structures

**DOI:** 10.3390/molecules29020505

**Published:** 2024-01-19

**Authors:** Liliya A. Yatsunyk, Stephen Neidle

**Affiliations:** 1 Department of Chemistry and Biochemistry, Swarthmore College, Swarthmore, PA 19081, USA; lyatsun1@swarthmore.edu; 2 UCL School of Pharmacy, University College London, London WC1N 1AX, UK

**Keywords:** left- and right-handed DNAs, quadruplex DNAs, crystal structures, water networks, spine of hydration, wheel of hydration

## Abstract

DNA requires hydration to maintain its structural integrity. Crystallographic analyses have enabled patterns of water arrangements to be visualized. We survey these water motifs in this review, focusing on left- and right-handed duplex and quadruplex DNAs, together with the i-motif. Common patterns of linear spines of water organization in grooves have been identified and are widely prevalent in right-handed duplexes and quadruplexes. By contrast, a left-handed quadruplex has a distinctive wheel of hydration populating the almost completely circular single groove in this structure.

## 1. Introduction

Biomolecules require a water environment to maintain their structural integrity, with the active involvement of those water molecules that are close to a protein or nucleic acid, which are less mobile than those in the bulk solvent. The role of water molecules in stabilizing and interacting with nucleic acid structures is long-established [1,2,3,4,5,6,7,8,9,10,11]. Water molecules serve to shield charge centers, such as phosphates, to bridge between DNA and ligands (both small molecules and proteins), and importantly to maintain the structural and conformational integrity of the DNA.

Seminal studies on the hydration of DNA fibers [12] first demonstrated the importance of hydration in maintaining the structural integrity of double-helical DNA as well as the role of hydration in determining its polymorphism, with the most notable polymorphs being the right-handed A- and B-forms [13,14]. However, these studies were unable to define the location of associated water molecules, although it was speculated that they are associated with phosphate groups on the exterior of the double helix [12]. Early single-crystal studies on nucleobases demonstrated the existence of direct base–water hydrogen bond contacts [15]. Subsequent single-crystal [16,17,18,19] and NMR [20] analyses of defined-sequence oligonucleotides and their drug complexes have revealed the role played by clusters of structured water molecules, establishing the importance of first- and second-shell waters. The “spine of hydration”, an array of relatively immobilized water molecules [16] present in the minor groove of AT-rich B-DNA, is perhaps the best-known water motif, with validation from high-resolution crystallographic studies as well as from NMR, simulations, and biophysical analyses (see, for example, Refs. [17,18,19,20,21,22,23,24,25,26,27,28]). Minor groove hydration can also play an active role in the recognition of small-molecule groove-binding ligands, with waters bridging between ligand and base atoms [29,30,31,32,33]. Hydrogen-bonded water pentagons and hexagons have been observed to occur in larger volume spaces such as in a DNA wide groove or at an intercalating drug–DNA interface (for example, Refs. [34,35]). 

DNA and RNA can also form three- and four-stranded structures, given appropriate sequences [36]. The latter, termed G-quadruplex (G4) nucleic acids, are of high current interest. They can be produced by the folding of a single DNA or RNA strand containing repeated short guanine (G) tracts, or by the association of two, three, or four such strands [36,37,38,39,40]. G4 sequences are widely, though non-randomly, prevalent in the genomes of many eukaryotic and prokaryotic organisms, including *Homo sapiens* [41,42,43,44], and the COVID-SARS2 virus [45]. Conventional wisdom is that the individual G tracts need to contain two or more guanine bases to effectively form sufficient G-quartets (≥2) for a stable G4 to be formed. Until recently, all G4s identified by structural methods (crystallography, NMR) and circular dichroism were right-handed (RH), in accord with most natural duplex DNAs and RNAs. This is the case whether G4 sequences are found in promoter or telomere regions, or in 5′/3′-UTR sequences. The importance of hydration in maintaining G-quadruplex structural integrity has been highlighted in several studies [46,47,48,49]. The hydration of high-resolution RH DNA G4s has been analyzed [50], and patterns of extended spines of groove waters have been observed in a groove of an antiparallel G4 structure, analogous to but distinct from the RH spine found in duplex B-DNAs. Detailed water arrangements in RH-G4s are dependent on G4 topology, with, for example, the presence of propeller loops preventing the formation of regular water spines but promoting local water clusters [50].

A novel class of G4 structures has recently been identified by crystallography and NMR; these G4 structures are left-handed [51,52,53,54,55] and are morphologically very distinct from the conventional RH parallel, antiparallel, or hybrid forms. These left-handed (LH) G4s have been derived from the aptamer AS1411 with the sequence [d(GGT)_4_TGT(GGT)_3_GG)] [56]. In solution, AS1411 forms a right-handed parallel topology G4 structure (note that the exact sequence of AS1411 was modified to include a thymine nucleotide added to the 5′- and 3′-ends of the sequence to facilitate homogeneity). The first LH-G4 structure to be determined [51] has the sequence d[T(GGT)_4_TG(TGG)_3_TGTT], and a minimal 12-nucleotide sequence for LH-G4 formation has been identified [54] as d[G(TGG)_3_TG], known as motif 1. Another minimal motif, motif 2, with the sequence d[(GGT)_3_GTG], was subsequently identified to also form an LH-G4 [52]. Both motifs contain three GG stretches and one “split” GG stretch; the latter is a requirement for LH-G4 formation. Structurally, LH-G4s contain two two-quartet domains and are either homo- or heterodimers of motifs 1 and 2 or structures formed by covalently connected motifs 1 and 2. A small number of putative left-handed sequences based on motifs 1 and 2 also occur naturally within the human genome, and presumably other genomes [51]. Their biological role is yet unknown. 

Because of the topological variability of RH-G4s, their four grooves can display greater variation in their geometries than those in duplex DNAs [57]. LH-G4 structures do not have the pattern of four external grooves found in RH-G4s; rather, they have one extended nearly circular groove between the two two-tetrad domains.

We report here on a survey that focuses on water arrangements found in crystal structures of LH-G4s and compare them with the arrangements in representative high-resolution LH duplex Z-DNA crystal structures, as well as in RH-G4s, i-motifs, and RH duplex B-DNA. This last category has been extensively described in the literature and will not be discussed here in detail. We highlight the unprecedented arrangement found in LH-G4s, of an extended two-layer water array in the form of a double left-handed ribbon filling the groove and making extended contacts with hydrogen bond donors and acceptors lining the walls of the groove. 

## 2. The Structures and Their Patterns of Hydration

### 2.1. RH-Duplex DNAs

**Figure 1:**
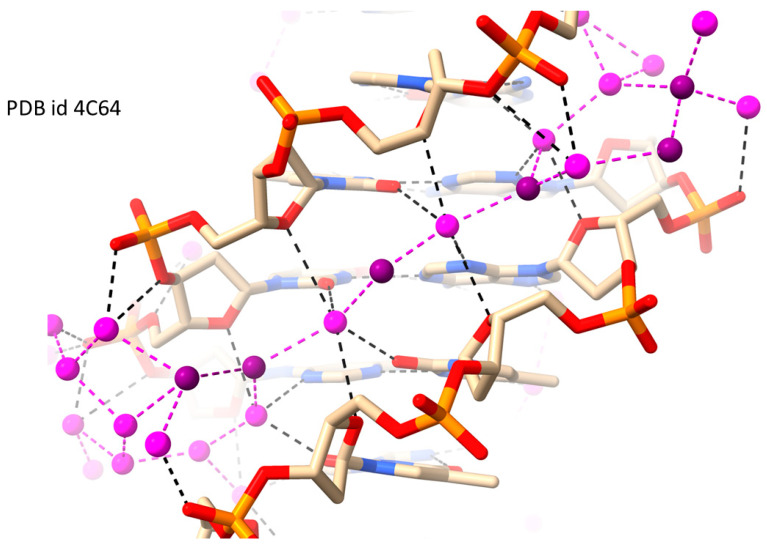
The classic minor groove spine of hydration in the high-resolution (1.32 Å) RH B-DNA crystal structure of PDB id 4C64 [58]. First-shell water molecules are colored mauve, and selected second-shell waters are colored dark purple.

The B-DNA polymorph, which is the most biologically relevant form of the DNA double helix, has a well-characterized structured water network in the minor groove (Figure 1: showing a typical high-resolution B-DNA dodecamer crystal structure [58]). This spine, in an AT-rich sequence, extends along the length of the sequence and thus follows the groove width which is narrowed at this sequence [14,15,16,17,18,19] and accommodates the string of waters, which is one molecule thick. Notably, the water molecules in the spine alternate between being part of the first and second hydration shells, so that any second-shell water is contacted by two first-shell waters, one on each side (Figure 1). These first-shell waters are involved in hydrogen bonds with A:T base pair edges lining the floor of the groove, and often additionally make hydrogen bond contacts with O4′/phosphate oxygen atoms lining the walls of the groove. 

### 2.2. LH Duplex DNAs (i.e., Z-DNA)

**Figure 2:**
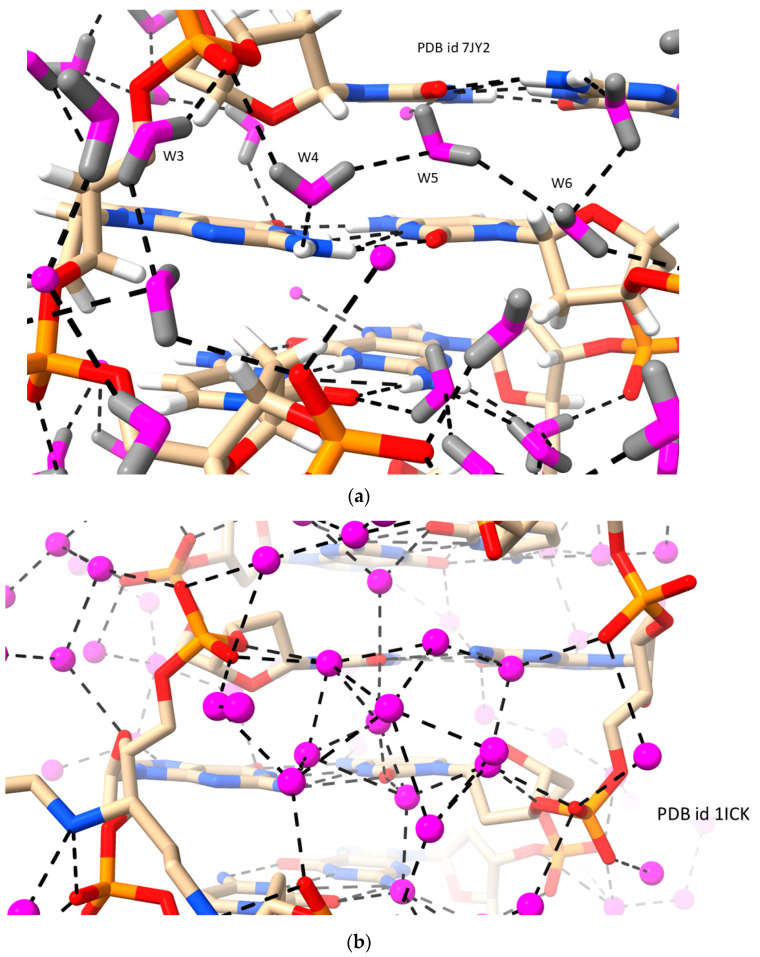
View of hydration in LH Z-DNA crystal structures. (**a**) A view of the hydration pattern in a high-resolution Z-DNA crystal structure of PDB id 7JY2 [8] determined by cryo-crystallography. Solvent oxygen atoms are colored magenta. Some of the solvent molecules are deuterated, and those deuterium atoms whose positions have been determined are colored grey. (**b**) A view of the dense cluster of water molecules in the minor groove region of a high-resolution Z-DNA structure of PDB id 1ICK [59].

Left-handed DNA duplexes in general have a sequence requirement of alternating C-G sequences, in contrast with the general sequence of B-DNAs. Many Z-DNA crystal structures have been determined, and their solvent structures have been analyzed (see, for example, Refs. [8,59,60,61,62]); of these Z-DNA crystal structures, a high proportion of the more recent high-resolution structures have significant solvent disorder (see, for example, Ref. [61]). The ultra-high resolution 0.55 Å crystal structure of the well-studied sequence (CGCGCG)_2_ [60] shows that the narrow Z-DNA minor groove is filled with inter-connected water molecules, connecting phosphate groups and base atoms on the deep minor groove floor (Figure 2a). Waters bridge between successive phosphate groups, and short water strings form inter-strand bridges between phosphate groups. First- and second-shell waters also form pentagonal and hexagonal water–phosphate–base hydrogen-bonded arrays. A deuterated water combined neutron/X-ray study [8] has revealed the orientation of deuterium atoms (Figure 2b) and a similar pattern of minor groove solvent bridging between phosphate groups. In general, the minor groove dimensions of Z-DNAs do not favor the continuous spine water arrangement seen in RH B-DNA sequences, and no comparable distinctive pattern has emerged. 

### 2.3. RH-G4s

The grooves in the majority of the known structurally simple RH-G4s are equivalent in terms of the character of their walls and floor, due to the symmetry of the G-quartet repeating unit, whose edges comprise the floor of each groove. Groove dimensions though may not be equivalent, due to the influence of loops. Parallel quadruplexes have all G-bearing strands oriented in the same direction, and all grooves tend to be of similar medium width. Three out of four G-bearing strands in hybrid structures are oriented in the same direction, so this category of G4 has three types of grooves, wide, medium, and narrow. Anti-parallel structures have two G-bearing strands oriented one way and two oriented in the opposite direction (for example, up–up–down–down in the dimeric *Oxytricha nova* G4 structure [63] or up–down–up–down in the human telomeric structure [64]) and two (wide and narrow) or three (wide, medium, narrow) types of grooves [50]. Groove dimensions are thus a consequence of strand polarity:

- Two adjacent strands running in the same direction generate a medium groove.
- Two adjacent strands running in opposite directions will generate either narrow or wide grooves.
- A narrow groove has all phosphate groups oriented into the groove; thus, in the adjacent groove, all phosphates on the shared strand between the two grooves are forced to be oriented away from the groove, making it either wide or medium but not narrow.
- Adjacent narrow grooves do not exist.
- Wide grooves have all phosphates oriented away from the groove.

Groove width determines whether a G4 groove contains continuous water organization, and phosphate orientations along the walls of the groove determine the hydrogen bond donation/acceptance pattern of the waters. Parallel G4s have their grooves interrupted by propeller loops, which tend to be positioned along the grooves, thereby inhibiting the formation of water-spine-like arrangements. Instead, high-resolution structures have revealed the presence of small discrete water clusters in these grooves [50].

**Figure 3:**
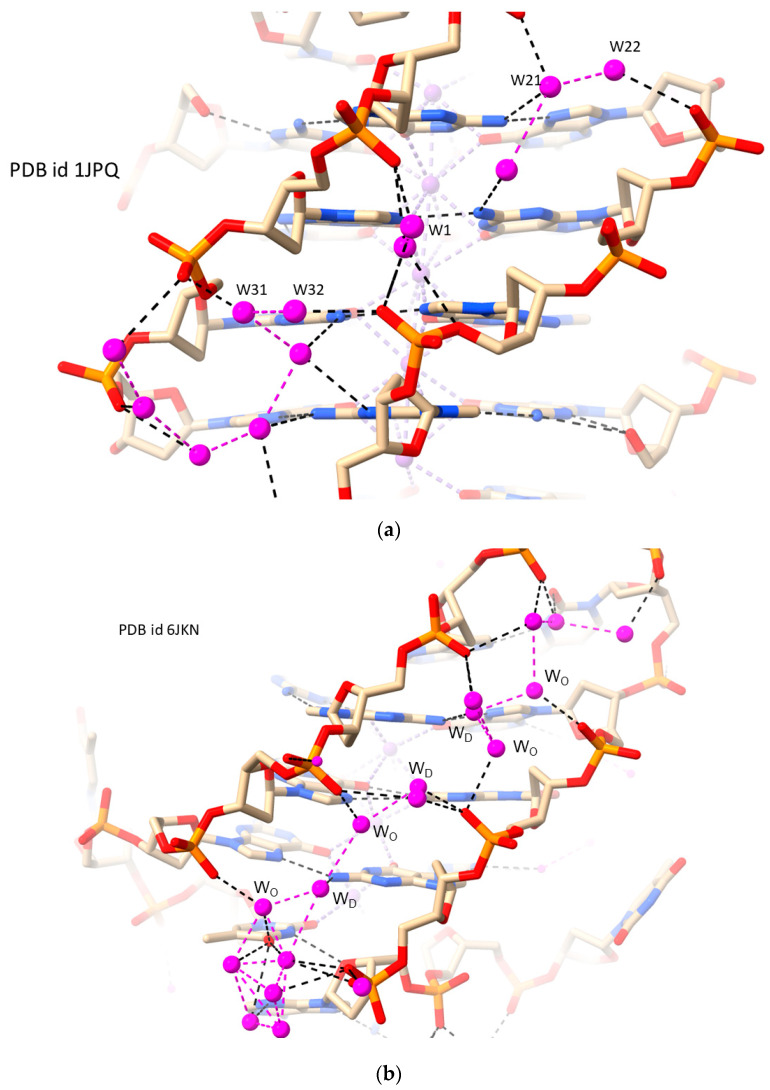

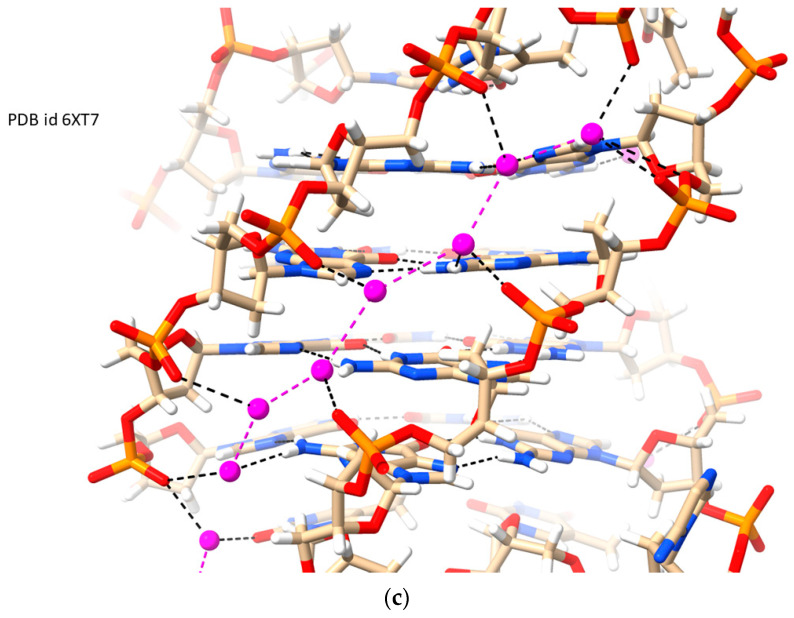
Views of the narrow groove hydration in three RH-G4 crystal structures. Water molecules are colored mauve. (**a**) PDB id 1JPQ [63]. Note that the waters do not form a continuous regular spine; although several waters contact phosphate groups, none contact O4′ sugar oxygen atoms. A water molecule (W1) in the center of the spine contacts both strands, where the groove is at its narrowest. Two pairs of waters (W21, W22; W31, W32) link phosphate groups on each strand. (**b**) The waters in a narrow groove of PDB id 6JKN [64] also form an irregular not perfectly continuous spine. Several “deep spine” (W_D_) and “outer spine” waters (W_O_) are highlighted. (**c**) The seven waters in the narrow groove of PDB id 6XT7 [66], by contrast, form a continuous and regular spine, with hydrogen bonding to phosphates, alternating from one strand to the other.

Antiparallel and hybrid G4s, on the other hand, have grooves that are of sufficient length for extended water arrangements to be formed. Figure 3a shows the almost continuous water spine in one of the two medium grooves (both of width 16.3 Å) in the antiparallel dimeric *Oxytricha nova* G4 structure [63]. At first glance, the spine appears closely similar to the RH B-DNA water spine [65] (Figure 1). However, it differs in the pattern of hydrogen bonding between DNA and the waters, since in the G4 the phosphate groups are oriented into the groove and thus can directly contact nearby waters. No hydrogen bonds with O4′ oxygen atoms are formed. Analogous to the RH B-DNA spine, waters also contact guanine base edge hydrogen bond donors/acceptors at the floor of the groove, although the pattern is less regular than that in the B-DNA arrangement [64,66]. Waters can also frequently bridge between strands (Figure 3b), and the groove is (or becomes) especially narrow in these regions [64]. The depth of this groove is such that there are in effect several layers of waters so that the spine is two-dimensional with “deep spine”, “mid spine”, and “outer spine” waters (Figure 3b). There is a second narrow water spine in one of these structures [63] (Figure 3c), that is unilayered and continuous.

### 2.4. RH i-Motifs

**Figure 4:**
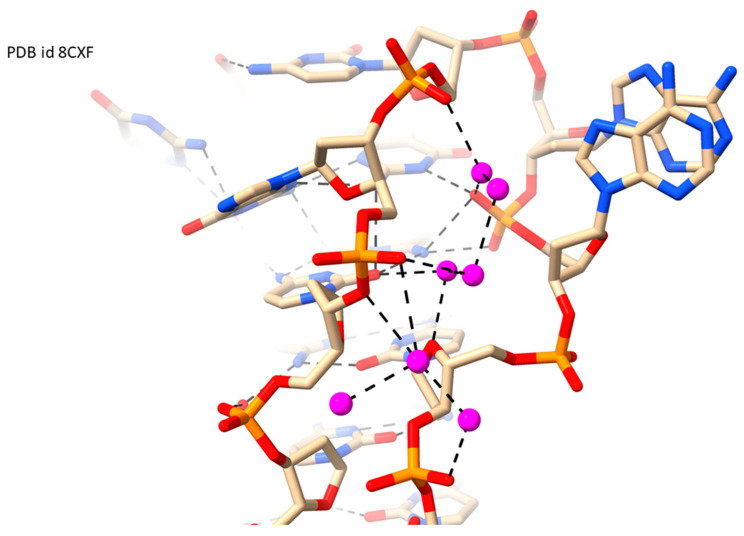
View into a narrow groove at one end of the i-motif crystal structure of PDB id 8CXF [67], showing water molecules colored magenta with hydrogen bonds as dashed lines to phosphate groups, sugar O4′, and cytosine base edges.

The i-motif is a four-stranded arrangement formed, ideally in low pH conditions, by the C-rich strand complementary to a G4-forming strand [65] or by an appropriate single-stranded C-rich sequence. The structures are characterized by two wide shallow grooves with two narrow grooves in between them. Crystal and NMR structures of several i-motif structures are available, although none of the currently available crystal structures are below 1.7 Å resolution, and none show extensive water networks. The highest-resolution one [67] does show evidence of a partial water network (Figure 4) in one of the minor grooves of this i-motif (encoded in the *HRAS* gene), suggesting that i-motif narrow grooves can support a spine-like water arrangement.

### 2.5. LH-G4s

**Figure 5:**
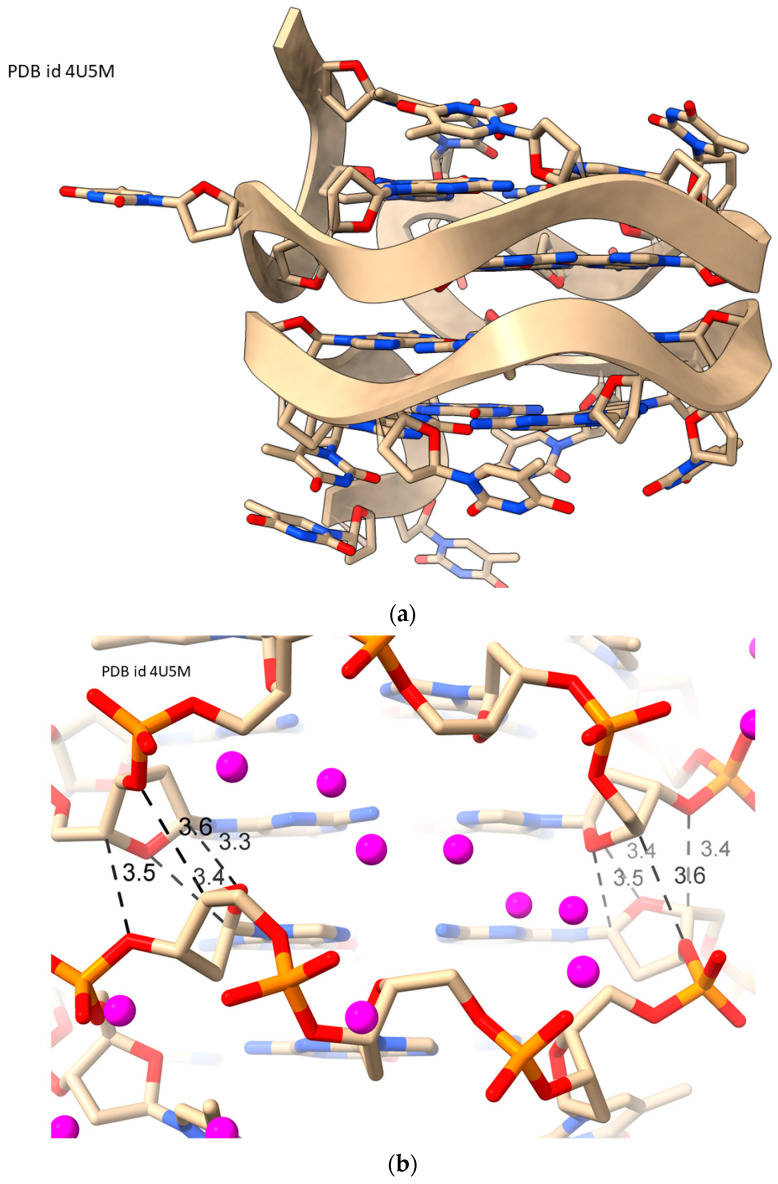
Views of LH-G4 structure PDB id 4U5M [51]. (**a**) Cartoon viewed along the G-quartet planes showing the single groove and its sinusoidal backbone character. (**b**) View into one of the wide groove regions, with waters colored in magenta and van der Waals distances highlighted on either side of the wide region; the upper and lower backbones become close, and the groove narrows.

In striking contrast to RH-G4s, there is only one groove, in the center of these structures. The deoxyfuranose-phosphodiester walls of the groove have a sinusoidal appearance (Figure 5a), with four alternating narrow and wide regions. The groove is parallel to the G-quartets and forms a continuous almost 360° wheel around the central G-quartet core. Each point of narrowing is caused by every fourth deoxyribose sugar in the groove, being in close van der Waals contact with the approximately 2-fold related deoxyribose on the opposite strand of the groove (Figure 5b). Atoms C1′, C4′, O3′, and O4′ of each deoxyribose are each packed ca. 3.4 Å separate from O4′, O3′, C4′, and C1′, respectively. 

**Figure 6:**
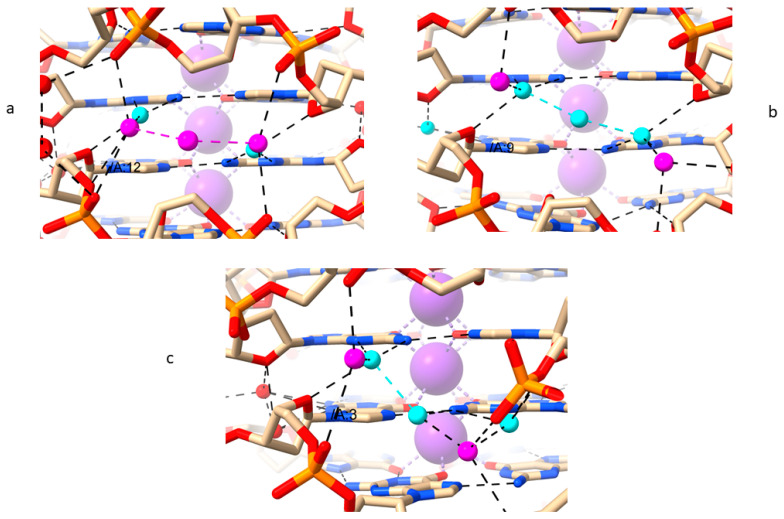
Three views of the water–G4 interactions in the LH-G4 structure of PDB id 4U5M [51]. Each of the views a-c is in one of the wider groove regions (labeled for convenience of referencing (**a**–**c**)) where the outer waters (colored mauve) form inter-strand hydrogen bonds and the inner ones (colored cyan) contact G-quartet base edges.

The three widest regions of the groove are between these deoxyribose pairs and are populated by a cluster of 5–6 water molecules (Figure 6). The intervening two nucleotides between each narrow groove region have their bases onto the end G-quartets or themselves are part of the end G-quartets. Both necessarily result in each intervening region having a pronounced groove bulge. The innermost shell of waters hydrogen bond to N2 and N3 of every guanine base edge forming the floor of the groove, alternating between the two G-quartets. Some of these water arrangements have pseudo-2-fold symmetry (Figure 6a,b in particular). It is notable that the water arrangements and the number of waters involved in each mini-cluster differ in detail, since the backbone geometry of each wide region is not precisely identical to its neighbor. Thus, although in most instances the outer waters form hydrogen bonds with phosphate oxygens and O4′ sugar atoms, the inner waters can also do so if the geometry permits (Figure 6b). The consistent pattern of O4′ sugar ring atoms hydrogen-bonded to the water clusters is in striking contrast to the lack of such contacts in RH-G4s. 

**Figure 7:**
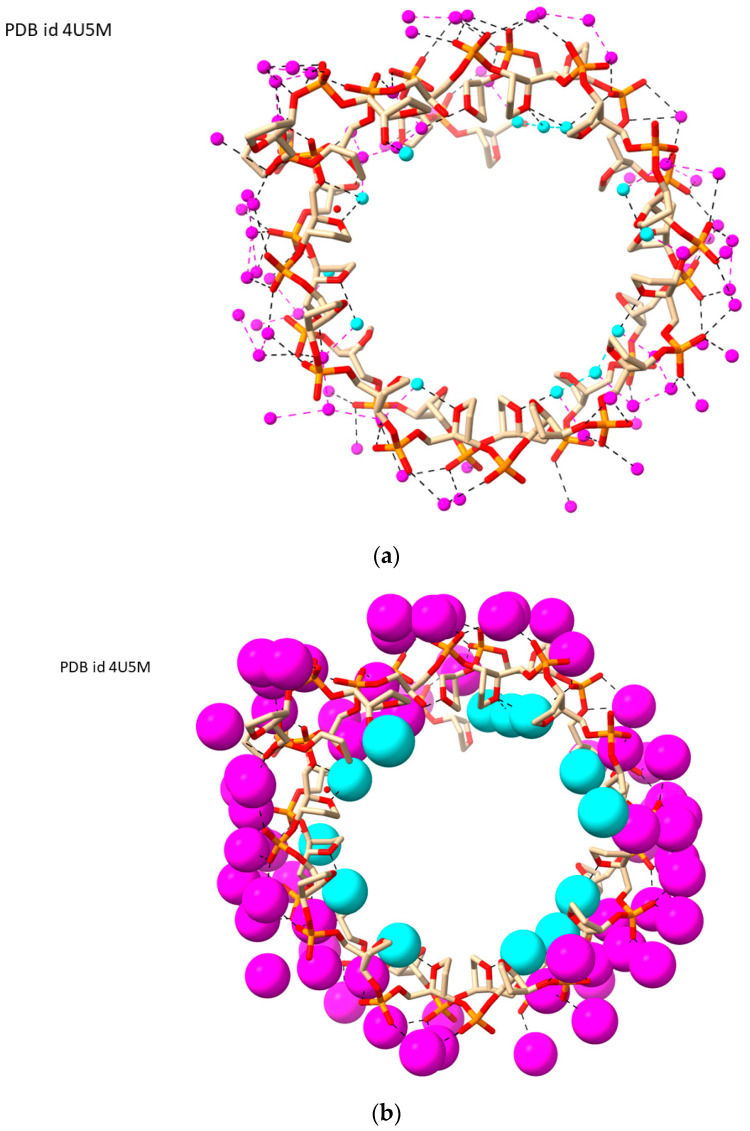
Views of LH-G4 structure of PDB id 4U5M [51], looking down the ion channel. Ions and guanine bases have been removed to enhance clarity. (**a**) View looking onto the G-quartet plane, although the G-quartets themselves have been removed for simplicity. Water molecules on the exterior of the G4 are shown as magenta spheres; those on the interior are colored cyan. (**b**) The same view of the structure, with the waters now shown as van der Waals spheres.

The water arrangement is not continuous around the groove, although in the absence of further structural data from more high-resolution structures, it is not clear whether this is real or a consequence of the lack of further water molecules being resolved in the crystal structure. The overall result is an almost complete wheel of hydration encircling the structure (Figure 7).

**Figure 8:**
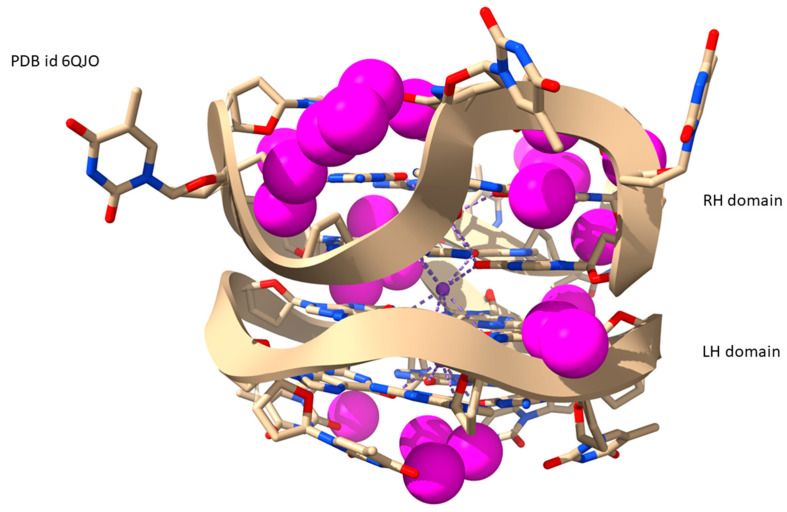
View of molecule A in the crystal structure of an LH-RH hybrid G4 of PDB id 6QJO [55], with water molecules shown as van der Waals spheres colored magenta.

The crystal structure of an LH-RH G4 hybrid [55] (Figure 8) also reveals a single groove around the structure and midway between the LH and RH domains. However, the groove is highly asymmetric, with deep loops protruding into the RH domain. These contain water molecules, although the resolution of the structure does not enable complete networks to be visualized. The NMR structure of the closely related sequence all-RH-G4 [56] also has a single groove, whose sinusoidal character closely resembles that of the LH-G4 described above, suggesting that the water wheel is not unique to this type of LH structure, but is more a consequence of the unusual G4 sequence type.

## 3. Discussion

This brief survey has highlighted the commonality of structured water assemblies in many LH and RH DNA duplexes and G-quadruplexes. Water assemblies are represented by water spines along extended grooves and water clusters, typically with pentagons and hexagons of waters connected to other waters and to the DNA backbone and/or base edges [50,65]. Water clusters are found in the B-DNA major groove and G4 loops and wide grooves.

Continuous grooves with unchanging width and depth typically contain connected extended spines of water molecules that are firmly hydrogen-bonded to groove walls and floors. This is the case for B-DNA (especially in narrow A/T regions) and in canonical G4s with antiparallel strands producing grooves of sufficient length. B-DNA spines have this A/T sequence requirement, whereas the presence of appropriate grooves in G4s is determined by topology. LH duplex DNA (i.e., Z-DNA) does not have the groove characteristics to produce water spines, even though there is extensive hydration in the grooves. LH-G4s have been reported, with a closely similar sequence and fold to a non-canonical RH-G4 aptamer sequence. Consequently, the nature of the single groove in these structures is similar, and it is reasonable to speculate that the wheel of hydration observed in the LH-G4 is also present in the RH version of the aptamer. Extended water spines are also likely to occur in G-wires: an atomic force microscopy study [68] indicates that the grooves are of appropriate dimensions. At present, we have no knowledge of whether the human genome or other genomes contain other categories of G4s in addition to the widely prevalent RH-G4 canonical ones [41,42,43,44] and the very rare LH-G4 circular type [51]. If more LH-G4 structures do exist, the nature of their grooves and consequent hydration patterns may well show novel features.

Water-filled cavities and grooves in DNAs are targets for small-molecule drugs, and X-ray studies have shown that waters are invariably found in the binding sites, often associated with bridging between ligands and DNA. This is the case in several B-DNA minor groove drug complexes [29,30,31] and the grooves/loops in some G4 drug complexes [69]. The observations of extended water spines in some G4s suggest that the design of appropriate G-bonding small molecules such as polyamides, to target these grooves and displace some bound water molecules, may be fruitful. They may also be of help in capturing otherwise transient topologies such as the LH-G4 one. 

**Figure 9:**
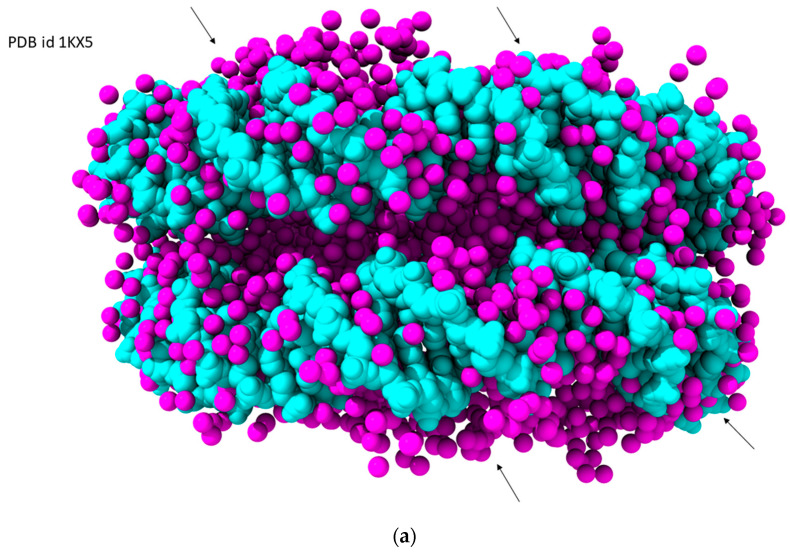

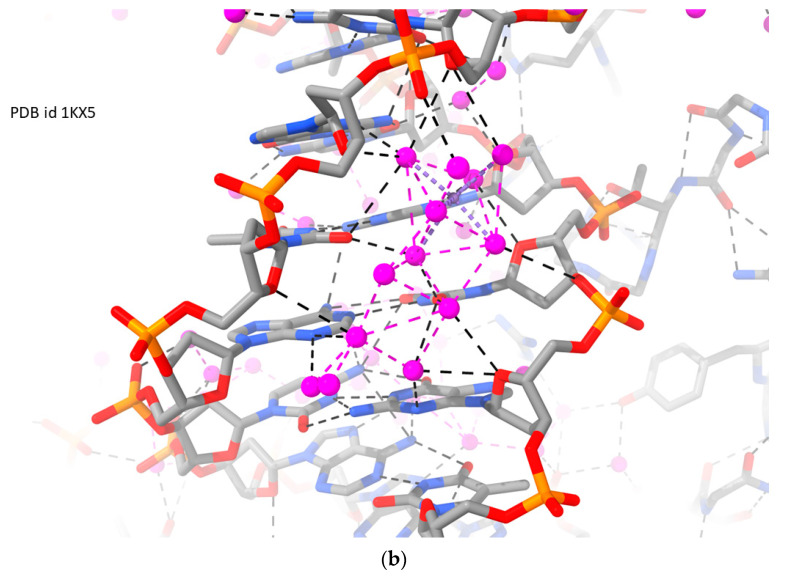
Views of the hydration network in the nucleosome core particle [72], PDB id 1KX5. (**a**) An overview of the complete 147-base-pair DNA structure, drawn in van der Waals space-filling mode and colored cyan. Histone proteins have been omitted for clarity. Water molecules are colored magenta. Four minor grooves are indicated by arrows. (**b**) A detailed view of one minor groove in the nucleosome structure, with water molecules colored magenta and hydrogen bonds with DNA and waters being shown by dashed lines.

Protein–DNA complexes are heavily hydrated [70,71]. Water molecules serve to shield charge centers, as well as helping to maintain DNA conformation and often bridging between protein residues and nucleotides. Analogous patterns of structured waters can be found in many protein–DNA crystal structures, although the perfect first- and second-shell patterns observed in native DNAs such as the spine of hydration can be modified depending on the individual site requirements. A notable example of water localization and analysis is in a nucleosome structure [72] at 1.9 Å resolution containing 147 DNA nucleotides (Table 1; Figure 9a). Over 3000 water molecules have been located in this study, of which 10% are in A/T-rich minor groove regions. Figure 9b shows the detailed hydration of one minor groove, which comprises several G:C base pairs and hence has been widened compared to a purely A/T stretch. The consequence of this widening is that the water arrangement is three-dimensional compared to the simpler one-dimensional spine observed in the A/T region of native DNAs. 

This review has focused on data from crystal structures, which conventionally represent time-averaged views. Even so, isotropic thermal parameters (B values, in Å^2^) of water molecules in high-resolution structures can provide insights into water motion. Unsurprisingly, first-shell waters in the minor groove tend to have lower mobilities as indicated by B and derived mean-square amplitude of vibration values (<u^2^>). Thus, in the spine of hydration in a high-resolution B-DNA crystal structure [52] (PDB id 4C64), the first-shell water molecules in the inner A/T region of the spine have a <B> of 19 Å^2^, whereas the second-shell waters have a <B> of 37 Å^2^. This significant difference is accentuated when comparing first- and outer-shell (i.e., semi-bulk) water molecules, although in general only a small proportion of these are reliably located in DNA crystallographic studies. Reduced water mobility (as shorter residence times) in the spine was reported in a seminal NMR study [20] and accords with more recent molecular dynamics simulations of the spine [11,28].

## 4. Methods

Individual crystal structures were taken from the PDB and chosen for analysis in this review (listed in Table 1). They have at least close to a full complement of first-shell water molecules around the DNAs. All analyses and visualizations of hydrogen-bonding distances and subsequent figure constructions were conducted with the ChimeraX program [73]. Donor–acceptor distances were accepted as hydrogen bonds if they were within the range 2.6–3.1 Å. Van der Waals distances were accepted within the range 3.4–3.6 Å.

## Figures and Tables

**Table 1:** Selected DNA crystal structures discussed in this review. Note that structure 7JY2 is from a combined X-ray/neutron diffraction study, with the 2nd set of resolution and R factor data referring to the neutron component. Structure 3P4J has 22 fully occupied water sites and 93 partially occupied water sites. Structure 1V9G is from a neutron diffraction study. Structure 6XT7 has four G4s in the crystallographic asymmetric unit, hence the large number of reported water molecules. Different structural categories have been highlighted in distinct colors. ^1^ The DNA in the nucleosome structure 1KX5 has the sequence d[TCAATATCCACCTGCAGATACTACCAAAAGTGTATTTGGAAACTGCTCCATCAAAAGGCATGTTCAGCTGGAATCCAGCTGAACATGCCTTTTGATGGAGCAGTTTCCAAATACACTTTTGGTAGTATCTGCAGGTGGATATTGAT].

Sequence	DNA Structure	Resolution (Å)	R Factor	No. of Solvent Atoms	PDB ID	Reference
d[CGCGAATTCGCG]	RH-B-DNA	1.32	0.139	131	4C64	[58]
Nucleosome core particle ^1^	RH-B-DNA	1.94	0.208	3130	1KX5	[72]
d[CGCGCG]_2_	LH-Z-DNA	1.00/1.50	0.156/ 0.277	77	7JY2	[8]
d[CGCGCG]_2_	LH-Z-DNA	0.95	0.086	83	1ICK	[59]
d[CGCGCG]_2_	LH-Z-DNA	0.55	0.068	78.6	3P4J	[60]
d[CGCGCG]_2_	LH-Z-DNA	1.80	0.222	44	1V9G	[61]
d[GGGGTTTTGGGG]	RH-G4 Antiparallel dimer	1.60	0.225	131	1JPQ	[63]
d[GGGTTAGG^Br^GTTAGGGTTAGG^Br^G]	RH-G4 Telomeric antiparallel	1.40	0.159	119	6JKN	[64]
d[G(TTGGGG)_4_]	RH-G4 Telomeric hybrid	1.56	0.154	379	6XT7	[66]
d[CGC_3_GTGC_3_TGCGC_3_GCAAC_3_GA]	RH i-motif	1.77	0.221	73	8CXF	[67]
d[(TGG)_4_TTG(TGG)_3_TGTT]	LH-G4	1.50	0.143	94	4U5M	[50]
d[GG(TGG)_2_TGTGTTGG(TGG)_3_TGTG]	LH-G4	1.69	0.20	80	7DFY	[51]
d[GT(GGT)_3_GTGGTGTGTGGTGG]	LH-G4 + 1 bulge	1.18	0.119	158	7D5D	[52]
d[GT(GGT)_3_GTGGTGTGTGTGTGG]	LH-G4 + 2 bulges	1.30	0.184	208	7D5E	[52]
d[G(TGG)_3_TG]	Minimal LH-G4	2.31	0.20	24	6FQ2	[53]
d[GT(GGT)_3_GTTGT(GGT)_3_GT]	LH-G4	2.01	0.185	10	6GZ6	[53]
d[GGTTGGTGTGGTTGGTTGT(GGT)_3_G	LH/RH-G4	1.80	0.164	166	6QJO	[54]
